# Postural Control and Muscle Activity during Dual-Task in Young Adults

**DOI:** 10.3390/bs14050403

**Published:** 2024-05-13

**Authors:** Marina Saraiva, João Paulo Vilas-Boas, Maria António Castro

**Affiliations:** 1 Dr. Lopes Dias Health School, Sector of Physiotherapy, Polytechnic Institute of Castelo Branco, 6000-767 Castelo Branco, Portugal; 2 RoboCorp Laboratory, i2A, Polytechnic Institute of Coimbra, 3046-854 Coimbra, Portugal; maria.castro@ipleiria.pt; 3 Centre for Mechanical Engineering, Materials and Processes, CEMMPRE, University of Coimbra, 3030-788 Coimbra, Portugal; 4 Faculty of Sports and CIAFEL, University of Porto, 4200-450 Porto, Portugal; jpvb@fade.up.pt; 5 LABIOMEP-UP, Faculty of Sports and CIFI2D, The University of Porto, 4200-450 Porto, Portugal; 6 School of Health Sciences, Sector of Physiotherapy, ciTechCare, CDRSP, Polytechnic University of Leiria, 2411-901 Leiria, Portugal

**Keywords:** dual task, muscle activity, postural stability, upright standing

## Abstract

In everyday life, we recurrently perform two tasks simultaneously, which is called dual-tasking. A common dual task is smartphone use while standing or walking. According to previous studies, this task can compromise postural stability. However, few studies have analyzed lower limb muscle activity during dual-tasking using smartphones. This study aimed to assess the postural sway and muscle activity during dual-tasking in young adults. Thirty-six healthy young adults (23.08 ± 3.92 years) participated in this study. They performed a single task (ST: keeping a quiet standing posture) and a dual task (DT: keeping the ST while simultaneously performing a cognitive task on their smartphone). Postural sway was assessed through the center of pressure (CoP) analysis using a force platform: total CoP displacement, CoP displacement in the anterior–posterior and medial–lateral directions, mean total velocity of the CoP, mean velocity of the CoP in the anterior–posterior and medial–lateral directions, and 95% confidence ellipse sway area. A surface electromyography system recorded the muscle activity of the lumbar spinal erector and five muscles of the lower limb (bilaterally). The results showed an increase in postural sway from the ST to the DT in all CoP variables (*p* < 0.05), and muscle activity in most muscles analyzed decreased from the ST to the DT (*p* < 0.05). In conclusion, our results reflect a decentralization of attention from motor performance once postural sway increased and muscle activity decreased in dual-task conditions.

## 1. Introduction

Maintaining balance in an upright standing posture seems like a simple activity. However, the capacity to stand is a complex task and is fundamental to initiating other activities, such as walking or running. Furthermore, it provides much information about the functionality of the postural control system. Postural control requires the interaction between the sensory, central nervous, and musculoskeletal systems to maintain static and dynamic postural stability [1], and the measure most utilized to assess balance performance during quiet standing is the center of pressure analysis using a force plate [2].

In daily life, we are constantly performing several tasks simultaneously. When we perform two tasks simultaneously, it is called a dual task. The dual-task paradigm can be used to determine the interference of concurrent tasks in motor and cognitive performance or to investigate the attentional demands of a motor task [3]. For example, a common task in our everyday lives is smartphone use, whether while standing or walking.

A narrative review analyzed previous studies that investigated the influence of smartphone use on postural control, essentially during gait, in which impairments in gait performance were reported, mentioning that few studies have assessed muscle activity [4]. In other dual-task conditions, a few studies have also evaluated muscle activity using surface electromyography (sEMG) [5,6,7,8]. Some studies have reported an increase in muscle activity, such as in the hamstrings, quadriceps, tibialis anterior, and gastrocnemius medialis, while performing a cognitive dual task compared with a single task [5,7]; others showed a decrease in muscle activity, like in the tibialis anterior, gastrocnemius, and soleus [6,8]. The studies that have assessed postural control in a quiet standing position while simultaneously performing tasks with a smartphone have shown decreased motor performance; consequently, postural stability deteriorated [9,10,11], which can contribute to the risk of injuries or falls. Moreover, previous studies have reported physical and psychological problems due to smartphone use [12,13] and increased pedestrian injury risk [14,15].

This study intends to add information about neuromuscular mechanisms involved in postural control through the center of pressure and muscle activity assessments, which can provide helpful information about muscles engaged in maintaining postural sway in dual-task conditions. Furthermore, to our knowledge, few studies have assessed muscular activity during postural tasks while simultaneously using a smartphone. Thus, given that smartphone use is more frequent among young adults [16], it is pertinent to carry out this study considering the fact that using a smartphone during postural or locomotor tasks causes disorders of balance and movement and less efficient static and dynamic postural control [17]. The present study investigated postural sway and muscle activity of the lower limbs and lumbar spinal erector through sEMG analysis in young adults performing cognitive–motor dual tasks. We hypothesized that muscle activity and the postural sway would differ between single and cognitive–motor dual tasks, indicating diminished static postural control in young adults under dual-task conditions.

## 2. Methodology

### 2.1. Participants

G*power software (Franz Faul, Edgar Erdfelder, Axel Buchner, Universität Kiel, Kiel, Germany, version 3.1.9.6) was used to calculate the sample size, considering an alpha level of 0.05, a power of 0.95, and a large effect size (Cohen’s *d* = 0.8), considering the study design. Thus, a minimum of twenty-four individuals were necessary.

We recruited thirty-six healthy young adults between 18 and 35 years old (23 male and 13 female) without any diseases or medication intake that would compromise task performance (see sample characteristics in Table 1). The sample was recruited through the dissemination of the study on social networks, and the volunteers contacted the researchers. The sample’s level of education corresponded to high school and university students. All participants gave their informed consent, and the Ethics Committee of the Polytechnic Institute of Coimbra approved the study (approval number: 27_CEPC2/2019).

### 2.2. Task Protocol

**Figure 1:**
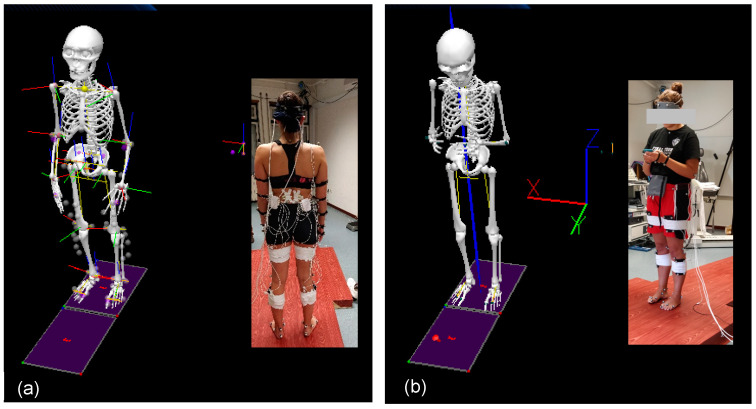
(**a**) Single-task and (**b**) dual-task performance.

All participants performed the following tasks while the CoP data and EMG were collected:

(1) Single task (ST—postural task): Young adults were instructed to maintain a relaxed standing position with the feet shoulder-width apart on the force platform, the arms along the trunk, and the eyes open, looking forward to a point at eye level [11] for 60 s (Figure 1).
(2) Cognitive–motor dual task (DT): Young adults were instructed to keep the standing position (ST) while simultaneously performing a cognitive task on a smartphone for 60 s (Figure 1).

The cognitive task consisted of performing subtraction and addition calculations with one or two digits or memorizing elements of various figures displayed on the smartphone screen. These tasks involve similar cognitive processes [18].

Cognitive task performance was calculated as the percentage of the correct responses given during cognitive tasks in a sitting position (cognitive single task: baseline cognitive task) and during the cognitive–motor dual task. All participants used their smartphones in the usual way and verbalized the answers. No task prioritization instructions were given.

### 2.3. Muscle Activity Collection and Analysis

EMG signals were recorded using a Bluetooth EMG system (PLUX, Lisbon, Portugal) following the SENIAM recommendations and protocols to prepare the skin and electrode placement [19]. Active surface Al/AgCl electrodes (rectangular shape, 30 mm × 22 mm) were placed to record EMG activity at a sampling frequency of 1000 Hz on the following postural muscles (bilaterally): biceps femoris (BF), rectus femoris (RF), tibialis anterior (TA), gastrocnemius medialis (GM), gastrocnemius lateralis (GL), gluteus maximus (GMax), and lumbar erector spinae (LES). These muscles were selected according to previous studies that measured muscle activity during static postural control [20,21,22].

EMG signals were amplified with a common-mode rejection ratio of 110 dB and an input impedance greater than 100 mV. Afterward, signals were digitally filtered between 20 and 490 Hz, smoothed through a low-pass filter at 12 Hz with a 4th Butterworth digital filter, and full-wave rectified. EMG was normalized on the basis of % MVC (maximal voluntary contraction), which was evaluated according to the procedures described by Konrad [23] and Hermens et al. [24]. The peak 200 ms EMG signal of the MVC was used as a reference for amplitude normalization. A MATLAB software (version R2020b, The Mathworks, Inc., Natick, MA, USA) routine was used for data processing, and the EMG average value during each task was calculated. All participants performed three maximal voluntary contractions for each muscle over 3 to 4 s, with two minutes of rest between repetitions, receiving verbal reinforcement to achieve the maximum contraction.

### 2.4. Center of Pressure Collection and Analysis

Postural sway was assessed through the center of pressure analysis using a force platform (model FP4060-07-1000, Bertec Corporation, 6171 Huntley Road, Suite J, Columbus, OH, USA). The following CoP variables were calculated: the total center of pressure displacement (TOTEX CoP), the center of pressure displacement in the anterior–posterior (AP-CoP) and medial–lateral (ML-CoP) directions, the mean total velocity of the CoP (MVELO CoP), mean velocity of the CoP in the anterior–posterior (MVELO CoP-AP) and medial–lateral (MVELO CoP-ML) directions, and the 95% confidence ellipse sway area (CEA). CoP data were filtered using a 50 Hz low-pass filter with 7th order Butterworth and processed through a MATLAB routine (version R2020b, The Mathworks, Inc., Natick, MA, USA).

### 2.5. Statistical Analysis

Descriptive statistics were performed to characterize the sample, and data were expressed as the mean ± standard deviation (SD). The normality of the distribution of the CoP and EMG data was verified using the Shapiro–Wilk test. The differences in postural sway and muscle activity between ST and DT were evaluated using the Wilcoxon signed-ranks test. These data were expressed as median values and interquartile ranges (IQRs). All statistical analyses were computed using IBM SPSS Statistics 25.0 software for Windows (SPSS, Inc., Chicago, IL, USA). The significance level was set at *p* < 0.05.

## 3. Results

### 3.1. Center of Pressure Behavior and Muscle Activity Pattern

**Figure 2:**
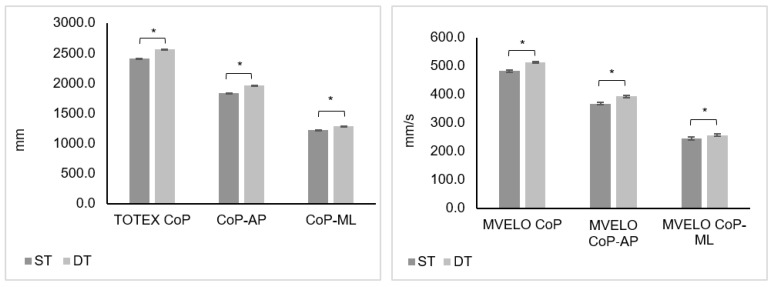
Comparison between postural sway during single and dual tasks, median values and standard errors (error bars). CoP, center of pressure; TOTEX CoP, total excursion of the center of pressure; CoP-AP, displacement of the center of pressure in anterior–posterior direction; CoP-ML, displacement of the center of pressure in medial–lateral direction; MVELO CoP, mean total velocity of CoP; MVELO CoP-AP, mean anterior-posterior velocity of CoP; MVELO CoP-ML, mean medial-lateral velocity of CoP. * *p*-value < 0.05 using Wilcoxon signed-ranks test.

Figure 2 shows the results found in the excursion and velocity parameters of CoP between the single and dual tasks. The total center of pressure displacement (ST: 2409.1 (2198.1–2806.0) mm vs. DT: 2567.4 (2374.7–3122.1) mm), the center of pressure displacement in the anterior–posterior (ST: 1837.6 (1650.9–2156.7) mm vs. DT: 1965.4 (1790.4–2362.4) mm) and medial–lateral (ST: 1224.7 (1076.5–1404.7) mm vs. DT: 1285.3 (1187.7–1512.8) mm) directions, the mean total velocity of the CoP (ST: 481.9 (439.7–561.3) mm/s vs. DT: 513.5 (475.0–624.5) mm/s); the mean velocity of the CoP in the anterior–posterior (ST: 367.5 (330.2–431.4) mm/s vs. DT: 393.1 (358.1–472.5) mm/s) and medial–lateral (ST: 245.0 (215.3–281.0) mm/s vs. DT: 257.1 (237.6–302.6) mm/s) directions increased from the single task to the dual task. Differences were found in all these variables between the single and dual tasks (*p* < 0.05).

There were differences found between the single- and dual-task conditions in the 95% confidence ellipse sway area (*p* < 0.001), with an increase from the single task (222.6 (113.5–364.6) mm^2^) to the dual-task condition (655.7 (303.2–1106.4) mm^2^).

All participants were asked which lower limb they would use to kick a ball to determine lower limb dominance. Most of the sample reported that their dominant lower limb was the right (94.4%), and while performing the dual task, they held the smartphone with both hands.

In the single task, no differences were found in muscle activity (% MVC) between each muscle’s left and right sides (TA, GM, GL, RF, BF, GMax, and LES: *p* > 0.05). On the other hand, in the dual task, there were differences in muscle activity (% MVC) between the left and right sides of each muscle analyzed (*p* < 0.05), in which muscle activity on the left side was greater than that on the right side.

Table 2 depicts the differences in muscle activity (% MVC) between the single and dual-task conditions. Muscle activity decreased in most muscles analyzed from the single task to the dual task. Differences between the ST and DT were found in the following muscles: the tibialis anterior and gastrocnemius medialis (bilaterally), the biceps femoris, the rectus femoris, the gastrocnemius lateralis, the gluteus maximus, and the lumbar erector spinae on the right side (*p* < 0.05). In the left side’s biceps femoris, gluteus maximus, and lumbar erector spinae increased their activity from the ST to the DT, and differences between tasks were found (*p* < 0.05). However, there were no differences in left biceps femoris and gastrocnemius lateralis activity between the single task and the DT (*p* > 0.05).

### 3.2. Cognitive Task Performance

Young adults increased their cognitive task performance from the cognitive single task (in the sitting position) to the cognitive–motor dual task. Differences were found (*p* = 0.003) between the percentage of the correct response given in the cognitive single-task (76.4 (50.5–91.3)%) and cognitive–motor dual-task (83.4 (60.6–92.5)%) conditions.

## 4. Discussion

This study analyzed the differences between the postural task (ST, quiet standing posture) and cognitive–motor dual task (DT, keeping a quiet standing position while simultaneously performing a cognitive task on a smartphone) in young adults.

There was an increase in all CoP parameters assessed (95% confidence ellipse sway area, total excursion, and velocity of the CoP mean value, as well as in the anterior–posterior and medial–lateral directions) from the ST to DT condition. These changes in CoP showed a decrease in postural control during cognitive–motor dual-task performance. In addition, the muscle activity of most muscles analyzed decreased from the ST to the DT, suggesting a decentralization of attention due to muscle relaxation in the DT condition. Furthermore, cognitive task performance increased from the cognitive single task to cognitive–motor DT condition, revealing that more attentional resources were allocated to cognitive tasks on the smartphone during the dual task than maintaining static postural control.

The increase in postural sway and decrease in the muscle activity of most muscles analyzed during dual-task performance, emphasizing decreased ankle muscle activity, suggests a change in postural control strategy from ST to DT conditions for maintaining balance. Furthermore, as the cognitive task performance increased from the sitting position to cognitive–motor DT and the postural sway increased from the ST to the cognitive–motor DT, we can suggest that young adults could be more concentrated on or motivated for cognitive task performance than motor task performance. 

A study showed a decline in tibialis anterior and gastrocnemius muscle response amplitude to a balance disturbance during the dual-task condition [6]. In this line, our results may indicate that with a decrease in muscle activity during a dual task, young adults may be less prepared to react to external perturbations due to the inadequate division of the cognitive resources available between cognitive tasks on a smartphone and maintaining a standing position. Furthermore, a previous study showed increased prefrontal cortex activity during dual tasking in young adults, demonstrating a prioritization of the cognitive task to the detriment of motor performance [25]. 

Under dual-task conditions, our results showed that EMG signals between the lower limbs’ left and right muscles differed, and the muscle activity in most muscles decreased from the ST to the DT. All of this, associated with increased postural sway, may indicate less efficient postural control performance when using a smartphone while standing due to inadequate central information processing of cognitive resources. According to theoretical approaches to dual-task interference [26], in this situation, the theory that can explain these alterations is the capacity sharing theory, in which the processing capacity is divided into two tasks, benefiting one task to the detriment of the other. On the other hand, factors like experience, age, motivation, task type, concentration, and fatigue can affect postural control [27,28].

The differences found between the muscular activity of the left lower limb and the right lower limb during the dual-task performance could be related to increased cognitive performance and the type of cognitive task used in the present study, which fits the role of the left prefrontal cortex [29]. Math tasks performed while standing can contribute to an increase in left dorsolateral prefrontal cortex activity [30], which can explain a greater decrease in right lower limb muscle activity compared with that on the left side due to a possible competition and sharing of neural resources and consequently less attentional resources available for an adequate efferent motor response.

Although the use of smartphones by young adults is widespread [16], this study shows that even under familiar and usual dual-task conditions, young adults reveal a limited capacity to allocate cognitive resources between two tasks that possibly share attentional resources [26,31].

Previous studies that assessed the influence of tasks using a smartphone while maintaining a standing posture showed that talking and texting on a smartphone affected postural stability in healthy young adults [11]. Another one concluded that performing a postural task and texting impairs postural stability [10]. The results of these studies are in line with ours (motor performance declines during dual-task conditions). However, the cognitive load of the secondary task used in the present study (memory and arithmetic tasks) may be greater than texting on a smartphone since texting is one of the tasks most used by young people on mobile phones [32]; perhaps for this reason, muscle activity also decreases due to the allocation of more attentional resources to cognitive tasks and a consequent decline in postural control. Although it is unclear, some studies have suggested that task difficulty can contribute to the decreased force produced and the recruitment of muscle motor units [6,33,34].

According to the conceptual model for characterizing patterns of cognitive–motor dual-task interference [35,36], young adults presented a cognitive priority trade-off strategy once the cognitive task performance improved while motor task performance decreased. Therefore, enhancing motor task performance during dual-task conditions is relevant to reducing other tasks’ cognitive interference and improving the capacity to react and adapt to postural perturbations. Therefore, dual-task training can be an appropriate intervention for improving postural stability [37], motor and cognitive performance [38], and reestablishing or enhancing the efficiency of allocating cognitive resources between two tasks performed simultaneously [6]. 

This study has a few limitations. First, considering the theoretical approaches to dual-task interference [26] and our results, it appears that young people allocate more attentional resources to cognitive tasks on smartphones than to postural control. However, this aspect could have been explored through the direct assessment of the cortical activity while young adults were performing the tasks with neuroimaging techniques, such as functional magnetic resonance imaging [39], electroencephalography [40], or functional near-infrared spectroscopy [41]. Thus, we could clarify the interference between the cognitive task and postural control during dual-task conditions. Finally, the positions of the head and upper limbs differed between the single and dual-task conditions, which could have affected the center of pressure behavior. Although the single task used was based on previous studies, we could have added a task in which the participants just simulated the position of holding the smartphone as if they were performing the cognitive–motor dual task. Thus, we could better explain if the position of the head and upper limbs influenced the center of pressure excursion.

We recommend future studies that use a similar methodology to investigate postural sway and muscle activity changes in other motor tasks, also using different challenge levels and in other age and pathologic groups.

## 5. Conclusions

Young adults performed a very familiar dual task in their daily lives; nonetheless, their motor performance during the dual task decreased compared with their motor performance during a single task. Under cognitive–motor dual-task conditions, postural sway increased and muscle activity decreased, indicating a decline in static postural control. This adaptative strategy may result from an inefficient allocation of cognitive resources when two tasks are performed concurrently, so clinical strategies to optimize or improve motor performance could be helpful.

## Figures and Tables

**Table 1:** Anthropometric and demographic characteristics of the sample (mean ± SD).

Variables	Sample n = 36
Age (years)	23.08 ± 3.92
Height (m)	1.71 ± 0.10
Body mass (kg)	73.99 ± 15.97
Body mass index (kg/m^2^)	25.15 ± 4.37

**Table 2:** Comparison between muscle activity during single and dual tasks, median values (IQR).

		Muscle Activity (sEMG)—% MVC		
		Single Task	Dual Task	*p*-Value ^1^
Tibialis Anterior	Left	11.30 (9.61–16.08)	5.49 (2.60–11.18)	0.001 *
	Right	13.60 (10.00–16.60)	1.30 (0.89–2.00)	<0.001 *
Gastrocnemius Medialis	Left	15.00 (12.63–22.88)	10.18 (5.88–14.60)	0.001 *
	Right	14.60 (11.80–19.85)	3.16 (2.21–5.41)	<0.001 *
Gastrocnemius Lateralis	Left	7.41 (4.96–10.88)	7.25 (4.18–16.43)	0.437
	Right	6.50 (5.10–10.60)	2.65 (1.91–3.92)	<0.001 *
Rectus Femoris	Left	3.81 (2.26–5.90)	5.63 (3.56–16.85)	0.008 *
	Right	3.84 (2.17–5.58)	2.59 (1.49–3.23)	<0.001 *
Biceps Femoris	Left	4.12 (2.97–7.16)	6.13 (3.83–12.65)	0.157
	Right	4.24 (2.63–7.24)	2.18 (1.16–3.83)	<0.001 *
Gluteus Maximus	Left	6.04 (4.27–9.34)	13.05 (7.50–23.53)	<0.001 *
	Right	5.97 (4.15–8.28)	4.32 (3.20–6.79)	<0.001 *
Lumbar Erector Spinae	Left	5.57 (4.23–7.38)	9.70 (4.64–14.88)	<0.001 *
	Right	5.86 (3.96–9.80)	4.94 (2.71–8.44)	0.001 *

sEMG, superficial electromyography activity; % MVC, percentage of maximum voluntary contraction. * *p*-value < 0.05 using; ^1^ Wilcoxon signed-ranks test.

## Data Availability

The available data are contained within the paper.
